# Fetal alcohol spectrum disorder: neurodevelopmentally and behaviorally indistinguishable from other neurodevelopmental disorders

**DOI:** 10.1186/s12888-019-2289-y

**Published:** 2019-10-28

**Authors:** Shannon Lange, Kevin Shield, Jürgen Rehm, Evdokia Anagnostou, Svetlana Popova

**Affiliations:** 1 Institute for Mental Health Policy Research, Centre for Addiction and Mental Health, 33 Russell Street, Toronto, ON M5S 2S1 Canada; 2 Institute of Medical Science, University of Toronto, 1 King’s College Circle, Toronto, ON M5S 1A8 Canada; 3 Factor-Inwentash Faculty of Social Work, University of Toronto, 246 Bloor Street, West, Toronto, ON M5S 1V4 Canada; 4 Dalla Lana School of Public Health, University of Toronto, 155 College Street, Toronto, ON M5T 3M7 Canada; 5 Institute of Clinical Psychology and Psychotherapy & Center for Clinical Epidemiology and Longitudinal Studies, Technische Universität Dresden, Chemnitzer Str. 46, 01187 Dresden, Germany; 6 Department of Psychiatry, University of Toronto, 250 College Street, Toronto, ON M5T 1R8 Canada; 7 Holland Bloorview Kids Rehabilitation Hospital, Bloorview Research Institute, 150 Kilgour Rd., East York, ON M4G 1R8 Canada; 8 Department of Pediatrics, University of Toronto, 555 University Avenue, Toronto, ON M5G 1X8 Canada

**Keywords:** Classification function, Fetal alcohol spectrum disorder, Neurodevelopmental profile

## Abstract

**Background:**

The lack of universally accepted diagnostic criteria and the high rate of psychiatric comorbidity make it difficult to diagnose Fetal Alcohol Spectrum Disorder (FASD). In an effort to improve the diagnosis of FASD, the current study aimed to identify a neurodevelopmental profile that is both sensitive and specific to FASD.

**Methods:**

A secondary analysis was conducted on data obtained from the Canadian component of the World Health Organization International Study on the Prevalence of FASD. Data on neurodevelopmental status and behavior were derived from a battery of standardized tests and the Child Behavior Checklist for 21 children with FASD, 28 children with other neurodevelopmental disorders, and 37 typically developing control children, aged 7 to 11 years. Two latent profile analyses were performed to derive discriminative profiles: i) children with FASD compared with typically developing control children, and ii) children with FASD compared with typically developing control children and children with other neurodevelopmental disorders. The classification function of the resulting profiles was evaluated using the sensitivity, specificity, positive predictive value (PPV), and negative predictive value (NPV). Confidence intervals (CIs) were approximated using 10,000 bootstrapped samples.

**Results:**

The neurodevelopmental profile of FASD tested consisted of impairments in perceptual reasoning, verbal comprehension, visual-motor speed and motor coordination, processing speed (nonverbal information), attention and executive function, visuospatial processing, and language, in combination with rule-breaking behavior and attention problems. When children with FASD were compared with typically developing control children, a 2-class model fit the data best and resulted in a sensitivity of 95.2% (95% CI: 84.2–100.0%), specificity of 89.2% (95% CI: 78.4–97.5%), PPV of 83.3% (95% CI: 66.7–96.2%), and NPV of 97.1% (95% CI: 90.3–100.0%). When children with FASD were compared with typically developing control children and children with other neurodevelopmental disorders, the neurodevelopmental profile correctly identified only 56.9% (95% CI: 45.1–69.2%) of typically developing children and children with other neurodevelopmental disorders as not having FASD, and thus the profile was found not to be specific to children with FASD.

**Conclusion:**

The findings question the uniqueness of children with FASD with respect to their neurodevelopmental impairments and behavioral manifestations.

## Background

Exposure to alcohol prenatally is the etiological cause of Fetal Alcohol Spectrum Disorder (FASD) – a term that is used to cover a range of diagnoses, including: Fetal Alcohol Syndrome (FAS), Partial FAS (pFAS), Alcohol-Related Neurodevelopmental Disorder (ARND), and depending on the diagnostic guideline, Alcohol-Related Birth Defects [1, 2]. Although historically used as a non-diagnostic umbrella term, it has recently been proposed that FASD be used as a diagnostic term with the specification of the presence or absence of sentinel facial features [3]. This is in agreement with the newly proposed diagnosis of Neurobehavioral Disorder Associated with Prenatal Alcohol Exposure (ND-PAE) in the Diagnostic and Statistical Manual of Mental Disorders, Fifth Edition (DSM-5) [4]. ND-PAE encompasses a range of neurobehavioral effects associated with prenatal alcohol exposure, and can be diagnosed independent of any physical findings [5].

It is well documented that individuals with FASD exhibit a broad array of neurodevelopmental impairments, such as deficits in adaptive function, attention, executive function, motor function, social cognition, verbal and nonverbal learning, as well as externalizing behaviors [6, 7]. It is also commonly reported that children with FASD have diminished intellectual functioning [8]; however, when compared with IQ-matched control children, differences in their neurodevelopmental presentation have been noted [9]. Although it is widely accepted that the neurodevelopmental and behavioral effects of prenatal alcohol exposure are far-reaching [7], the current diagnostic guidelines tend to focus on the severity of the neurodevelopmental impairments rather than on the specific impairments.

Early and accurate diagnosis of FASD is crucial to providing timely developmental interventions, which are key to altering the developmental trajectory of affected individuals with respect to social functioning, improving their quality of life, and preventing subsequent adverse outcomes common among individuals with FASD, such as school failure and dropping out, addiction, mental health problems, dependent living, as well as involvement with the law and incarceration [10]. However, even in clinical settings where FASD is an important area of emphasis, individuals who have been affected by prenatal alcohol exposure often go undiagnosed or are misdiagnosed [11]. This can likely be attributed to the fact that the diagnosis of FASD is complicated due to difficulties in obtaining confirmation of prenatal alcohol exposure, a high rate of psychiatric comorbidity [12, 13], and the existence of signs and symptoms that overlap with those of other neurodevelopmental disorders [14].

Thus, with the aim of improving screening and diagnostic efforts, the concept of a unique neurodevelopmental profile of FASD, defined as the outward expression (behavioral and developmental) of the central nervous system damage caused by prenatal alcohol exposure, has received some attention in recent years. A distinct neurodevelopmental profile of FASD could assist in accurately identifying individuals with FASD, distinguishing between FASD and other conditions that present similar clinical features, improving clinical services for individuals with FASD, and triaging of individuals most in need of a full multidisciplinary FASD diagnostic assessment. Further, a unique neurodevelopmental profile of FASD could aid in the ascertainment of accurate prevalence estimates, as well as the planning and development of appropriate targeted interventions for individuals with FASD. Therefore, the objective of the current study was to identify a neurodevelopmental profile that is both sensitive and specific to FASD.

As discussed above, FASD includes several distinct diagnoses. As such, there is the possibility that individuals with FASD exhibit more than one neurodevelopmental profile (i.e., a unique profile could exist for each diagnostic category). In order to explore this possibility, the current study utilized a methodology that allows for the empirical determination of the number of distinct profiles.

## Methods

### Participants

This study was a secondary analysis of data for 37 typically developing children (70.3% male; mean [standard deviation (SD)] age: 9.0 [1.0] years), 21 children with FASD (52.4% male; mean [SD] age: 9.7 [0.8] years), and 28 children with other neurodevelopmental disorders (Attention Deficit Hyperactivity Disorder [ADHD] and/or Autism Spectrum Disorder [ASD]; 75.0% male; mean [SD] age: 9.3 [1.0] years) from the Canadian component of the World Health Organization (WHO) International Study on the Prevalence of FASD [15]. The Canadian FASD prevalence study employed a cross-sectional, observational design using active case ascertainment, along with retrospective collection of prenatal alcohol exposure information, to identify cases of suspected FASD among elementary school students in grades 2, 3, and 4 attending public school in the Greater Toronto Area in Ontario, Canada. The study procedures followed a step-wise approach, where only those students meeting predetermined criteria proceeded to the subsequent phase. Phase I involved: 1) taking growth measurements, 2) identifying learning and/or behavioral problems, and 3) a dysmorphology examination. Phase II involved: 1) a neurodevelopmental assessment, 2) maternal interview, and 3) behavioral observations/ratings by parents, obtained via the Child Behavior Checklist (CBCL) [15]. In addition, a group of typically developing control children was randomly selected from a list of all students who completed Phase I and who did not meet the criteria for Phase II using a systematic sampling technique; these students underwent a complete assessment in Phase II. Final diagnostic screening conclusions were made, by consensus, by a team of experienced multidisciplinary experts, using the 2005 Canadian diagnostic guidelines [1]. A detailed description of the methodology of the Canadian FASD prevalence study is presented in Popova et al. [16].

### Measures

#### Neurodevelopmental assessment

Neurodevelopmental assessments were conducted by qualified psychometrists using the WHO International Study on the Prevalence of FASD test battery, which included: Wechsler Abbreviated Scales of Intelligence, Second Edition (WASI-II [17]; subtests administered included block design, matrix reasoning, similarities, and vocabulary); Wechsler Intelligence Scale for Children, Fourth Edition (WISC-IV [18]; subtests administered included coding, digit span, symbol search, and letter-number sequencing); and NEPSY, Second Edition (NEPSY-II [19]; subtests administered included arrows, auditory attention, fingertip tapping, response set, and word generation). This test battery was devised based on the minimum measurements necessary to screen children for FASD, as per expert opinion. All tests were administered and scored by the examiner according to published test manuals and rechecked by a second examiner. Raw scores were converted to scaled scores according to age and sex norms. Canadian norms were used for the WISC-IV, and US norms were used for the WASI-II and NEPSY-II (as Canadian norms are not available for the respective instruments).

#### Behavioral observations/ratings by parents

Parents were asked to complete the CBCL to evaluate their child’s social competencies and identify any behavioral problems. The CBCL is a widely used, standardized questionnaire to assess emotional and behavioral problems in children aged 6–18 years [15]. T-scores were computed for 23 composite scales using 113 behavioral descriptors, scored on a three-point Likert scale (0 = not true, 1 = somewhat or sometimes true, 2 = very true or often true), according to age and sex norms.

### Latent profile analysis

#### Analysis 1: children with FASD vs. typically developing control children

Latent profile analysis (LPA) [20] was first performed on a sample of children with FASD and typically developing control children in order to identify the measures that best differentiate the two groups of participants. A step-wise approach was used to select indicator variables for the LPA. A total of 42 variables were available for consideration (22 derived from the subtests of the neurodevelopmental test battery and 20 derived from the composite scales of the CBCL). Variables were initially selected based on standardized differences in means between children with FASD and typically developing control children (measured through Cohen’s *d*) [21]; variables with a large effect size (*d* ≥ 0.8) were retained. Pearson’s correlation coefficients (*r*) were calculated to avoid the inclusion of redundant variables. For strongly correlated variables (*r* ≤ − 0.7 or *r* ≥ 0.7), the variable with the larger effect size was retained. If the effect sizes were equal, Student’s unpaired t-tests were performed (to test differences in the means between children with FASD and typically developing control children), and the variable with the larger t-score was retained.

##### Post-hoc analysis

High levels of prenatal alcohol exposure have been found to be associated with an increased risk of impaired intellectual functioning [8]. In order to determine if children with FASD are distinguishable from typically developing control children by IQ alone, LPA was performed using IQ (i.e., the WISC-IV, FSIQ-4 score) only.

#### Analysis 2: children with FASD vs. typically developing control children and children with other neurodevelopmental disorders

To determine whether the neurodevelopmental profile identified in analysis 1 is specific to FASD, LPA was performed on the complete sample (i.e., children with FASD, children with other neurodevelopmental disorders, and typically developing children). In addition to the class solution selected based on model fit, as described below, a 4-class model was also explored as there were four diagnostically distinct groups included in the sample (FASD, ADHD, ASD, and typically developing children).

##### Sensitivity analysis

Given the small number of children with ASD in the group of children with other neurodevelopmental disorders and the shared characteristics of FASD and ASD [22], LPA was performed on a sample of children with FASD (*n* = 21), children with ADHD (*n* = 22), and typically developing control children (*n* = 37) to determine the influence that the inclusion of children with ASD had on the ability of the neurodevelopmental profile to differentiate the respective groups of children.

#### Model selection

The number of subgroups in the sample was tested iteratively based on the following model fit statistics: Akaike Information Criterion (AIC) [23], Bayesian Information Criterion (BIC) [24], log likelihood, and the Lo-Mendell-Rubin adjusted log likelihood ratio test [25]. Optimal model fit was defined by lower relative AIC and BIC values and higher log likelihood values. Further, an entropy value > 0.8 was used as an indicator of highly discriminating latent classes (i.e., an indicator of low classification uncertainty) [26].

#### Model evaluation

In LPA, following the determination of the likely number of classes, participants were subsequently assigned to a subgroup based on the probability of membership as indicated by the model. This assignment allows for the model’s classification function, as a binary classification test, to be evaluated. This evaluation was achieved through the calculation of the resulting model’s sensitivity, specificity, positive predictive value (PPV), and negative predictive value (NPV). The 95% confidence interval (CI) for each measure of interest was approximated using the 2.5th and 97.5th percentiles of 10,000 bootstrap generated estimates. Further, Cohen’s *d* and unpaired Student’s t-test for normally distributed data were used to compare the resulting subgroups on each of the observed indicator variables.

### Missing data imputation

Little’s missing completely at random (MCAR) test [27] was performed to test the assumption that missing data were missing completely at random (*X*^*2*^*(3)* = 2.575, *p* = 0.462); this was confirmed. As such, missing data (0.5% of the data were missing) were replaced by the mean score of the complete cases in the study sample (i.e., mean imputation).

### Statistical software

Variable selection and the MCAR test were performed using Stata version 15.1 [28], the LPA was conducted using Mplus version 8.0 [29], and CIs were computed in R version 3.4.4 [30]. Statistical significance was based on an acceptable type-I error rate (α) of 0.05.

## Results

The three groups of children differed from one another with respect to ethnicity (*p* = 0.020), height ≤ 10th percentile (*p* = 0.041), and occipitofrontal circumference ≤ 10th percentile (*p* = 0.011). With respect to mean IQ, both children with FASD (mean = 87.2 [SD = 10.2]) and children with other neurodevelopmental disorders (mean = 95.6 [SD = 14.1]) differed from typically developing control children (mean = 106.4 [SD = 12.9]; *p* = < 0.001 and *p* = 0.003, respectively), but were not significantly different from one another (*p* = 0.064). The groups did not significantly differ from one another on age, sex, handedness, weight ≤ 10th percentile, or the three characteristic facial features that discriminate individuals with and without FAS or pFAS (i.e., palpebral fissure length 2 standard deviations below the mean, smooth philtrum, and thin vermillion border). Demographic and descriptive data for study participants are presented in Table 1.

**Table 1:** Demographic and descriptive characteristics of study participants

	Typically developing control children (*n* = 37)	Children with FASD (*n* = 21)	Children with other neuro-developmental disorders (*n* = 28)	Statistical test	*P*-value^a^
Age (years) – mean (SD)	9.0 (1.0)	9.7 (0.8)	9.3 (1.0)	*F* = 3.63	0.505
Range	7.2–11.3	7.9–11.0	7.3–10.7		
Sex (male) – n (%)	26 (70.3)	11 (52.4)	21 (75.0)	*X* = 3.03	0.220
Handedness (right) – n (%)	32 (86.5)	17 (81.0)	28 (100.0)	*X* = 5.29	0.071
Ethnicity – n (%)				*X* = 24.09	0.020
Caucasian	16 (43.2)	15 (71.4)	13 (46.4)		
African Canadian/Caribbean	0 (0.0)	1 (4.8)	4 (14.3)		
Eastern European	5 (13.5)	2 (9.5)	0 (0.0)		
Western European	11 (29.7)	3 (14.3)	2 (7.1)		
Chinese/South East Asian	1 (2.7)	0 (0.0)	5 (17.9)		
South Asian/Other	4 (10.8)	0 (0.0)	4 (14.3)		
IQ^b^ – mean (SD)	106.4 (12.9)	87.2 (10.2)	95.6 (14.1)	*F* = 16.18	< 0.010^c,d^
Range	80–138	71–116	63–120		
Height ≤ 10th percentile – n (%)	1 (2.7)	5 (23.8)	3 (10.7)	*X* = 6.37	0.041
Weight ≤ 10th percentile – n (%)	6 (16.2)	4 (19.1)	4 (14.3)	*X* = 0.20	0.905
OFC ≤10th percentile – n (%)	0 (0.0)	5 (23.8)	5 (17.9)	*X* = 8.96	0.011
Right PFL 2SD below the mean – n (%)	8 (21.6)	10 (47.6)	11 (39.3)	*X* = 4.63	0.099
Left PFL 2SD below the mean – n (%)	9 (24.3)	9 (42.9)	9 (32.1)	*X* = 2.15	0.342
Smooth philtrum (4 or 5 on the Lip-Philtrum Guide) – n (%)	12 (32.4)	5 (23.8)	9 (32.1)	*X* = 4.78	0.572
Thin vermillion border (4 or 5 on the Lip-Philtrum Guide) – n (%)	8 (22.2)	4 (19.1)	3 (10.7)	*X* = 3.77	0.708
FASD diagnostic category^e^ – n (%)					
FAS		3 (14.3)			
pFAS		2 (9.5)			
ARND		16 (76.2)			
Other neurodevelopmental disorders^f^ – n (%)					
ADHD		5 (23.8)	23 (82.1)		
ASD		3 (14.3)	6 (21.4)		

*ADHD* Attention Deficit Hyperactivity Disorder, *ARND* Alcohol-Related Neurodevelopmental Disorder, *ASD* Autism Spectrum Disorder, *FAS* Fetal Alcohol Syndrome, *FASD* Fetal Alcohol Spectrum Disorder, *OFC* Occipitofrontal Circumference, *pFAS* Partial Fetal Alcohol Syndrome, *PFL* Palpebral Fissure Length, *SD* Standard Deviation

^a^*p*-values are based on chi-square (for categorical variables) and Student’s unpaired t-test (for continuous variables). ^b^WISC-IV: FSIQ-4. ^c^Children with FASD vs. Typically developing control children. ^d^Children with other neurodevelopmental disorders vs. Typically developing control children. ^e^As per the 2005 Canadian diagnostic guidelines [1]. ^f^Not mutually exclusive

### Analysis 1: children with FASD vs. typically developing control children

Based on the variable selection process, described above, ten observed indicator variables were retained. Eight variables were derived from the neurodevelopmental test battery (WASI-II - block design, similarities, and vocabulary; WISC-IV - coding and symbol search; and NEPSY-II - response set, arrows, and word generation (letters)), and two variables were derived from the CBCL (attention problems and rule breaking behavior). Based on the model fit statistics, a 2-class model best fit the data (see Table 2 for the model fit statistics).

**Table 2:** Model fit statistics for analysis 1 and 2

Model fit statistics	Analysis 1					Analysis 2						
	Children with FASD vs. Typically developing control children			*Post-hoc analysis*^b^*:* Children with FASD vs. Typically developing control children		Children with FASD vs. Typically developing control children and children with other neurodevelopmental disorders				*Sensitivity analysis:* Children with FASD vs. Typically developing control children and children with ADHD		
	1-class	2-class	3-class	1-class	2-class	1-class	2-class	3-class	4-class	1-class	2-class	3-class
AIC	3275.21	3159.45	3144.57	482.44	483.83	4850.13	4693.50	4665.17	4635.78	4504.74	4358.25	4335.97
BIC	3316.42	3223.33	3231.11	486.56	492.07	4899.22	4769.58	4768.25	4765.86	4552.38	4432.09	4436.02
LLV	− 1617.60	− 1548.73	− 1530.29	− 239.22	− 237.91	− 2405.07	− 2315.75	− 2290.58	− 2264.89	− 2232.37	− 2148.13	− 2125.99
*p*-value^a^	–	0.149	0.512	–	0.190	–	0.002	0.265	0.422	–	0.008	0.391
Entropy	–	0.903	0.956	–	0.682	–	0.894	0.924	0.924	–	0.894	0.926

*AIC* Akaike information criterion, *BIC* Bayesian information criterion, *LLV* Log-likelihood value, “-” Not applicable

^a^*p*-value is in reference to the respective model’s comparison with the lower class solution

^b^Model with only IQ as an indicator variable

In this model, 24 participants (41.4% of the sample) were assigned to subgroup 1, and 34 participants (58.6% of the sample) were assigned to subgroup 2. Participants in subgroup 1 performed worse than participants in subgroup 2 for each of the above eight observed variables derived from the subtests of the neurodevelopmental test battery and scored higher on the above two observed variables derived from the composite scales of the CBCL (Table 3).

**Table 3:** Mean scores for each subgroup in the 2-class models in analysis 1 and 2

Observed variable/measure	Analysis 1 FASD vs. Typically developing control children							Analysis 2 FASD vs. Typically developing control children and children with other neurodevelopmental disorders						
	Subgroup 1^a^		Subgroup 2^a^		*d*	t-score	*p*-value	Subgroup 1^b^		Subgroup 2^b^		*d*	t-score	*p*-value
	Mean	SD	Mean	SD				Mean	SD	Mean	SD			
Neurodevelopmental Test Battery														
WASI-II: block design	8.79	2.62	12.32	3.88	1.03	4.135	< 0.001	9.17	2.79	12.63	3.71	1.07	4.785	< 0.001
WASI-II: similarities	7.79	2.57	10.47	3.22	0.99	3.863	< 0.001	8.08	2.35	10.63	3.09	0.94	4.212	< 0.001
WASI-II: vocabulary	7.96	3.18	12.00	3.70	1.16	4.450	< 0.001	8.27	3.01	12.11	3.55	1.18	5.311	< 0.001
WISC-IV: coding	5.63	3.44	10.15	2.35	1.59	5.590	< 0.001	5.75	3.08	10.58	2.15	1.78	8.549	< 0.001
WISC-IV: symbol search	6.79	2.47	11.59	2.58	1.89	7.152	< 0.001	7.08	2.41	11.68	2.64	1.83	8.331	< 0.001
NEPSY-II: arrows	7.25	3.23	11.59	2.45	1.55	5.544	< 0.001	7.75	3.31	11.87	2.03	1.46	7.098	< 0.001
NEPSY-II: response set	7.13	3.48	11.82	2.79	1.52	5.485	< 0.001	7.91	3.29	12.03	2.87	1.32	6.191	< 0.001
NEPSY-II: word generation (letter)	8.08	3.23	10.37	2.63	0.79	2.861	0.006	8.29	2.94	10.72	2.52	0.88	4.127	< 0.001
Child Behavior Checklist														
Attention problems	60.73	8.08	51.53	2.30	1.68	5.421	< 0.001	62.38	8.22	53.13	5.16	1.32	6.374	< 0.001
Rule breaking behavior	56.08	6.67	51.88	2.96	0.87	2.885	0.007	56.50	7.19	52.76	4.03	0.62	3.042	0.003

*d* Cohen’s *d*, *SD* Standard deviation

^a^Subgroup 1 is comprised of 20 children with FASD and four typically developing control children; and Subgroup 2 is comprised of one child with FASD and 33 typically developing control children

^b^Subgroup 1 is comprised of 20 children with FASD, 22 children with other neurodevelopmental disorders, and six typically developing control children; and Subgroup 2 is comprised of one child with FASD, six children with other neurodevelopmental disorders, and 31 typically developing control children

The final 2-class model resulted in 91.4% of participants being classified correctly overall, with almost all (20 out of 21; 95.2% [sensitivity], 95% CI: 84.2–100.0%) children with FASD assigned to subgroup 1, and notably more (33 out of 37; 89.2% [specificity], 95% CI: 78.4–97.5%) typically developing control children assigned to subgroup 2 (Table 4).

**Table 4:** Number of participants assigned to each subgroup and the classification function of the 2-class model in analysis 1

Group assignment; Classification function^a^ (%)	Children with FASD (n)	Typically developing control children (n)
Subgroup 1	20	4
Subgroup 2	1	33
Sensitivity (%)	95.2 (95% CI: 84.2–100.0)	
Specificity (%)	89.2 (95% CI: 78.4–97.5)	
PPV (%)	83.3 (95% CI: 66.7–96.2)	
NPV (%)	97.1 (95% CI: 90.3–100.0)	

*CI* Confidence Interval, *FASD* Fetal Alcohol Spectrum Disorder, *n/a* Not Applicable, *NPV* Negative Predictive Value, *PPV* Positive Predictive Value

^a^Assuming subgroup 1 is reflective of the neurodevelopmental profile of FASD and subgroup 2 is reflective of typically developing control children

#### Post-hoc analysis: latent profile analysis based on IQ only

When IQ was included as the only indicator variable, a 1-class model fit better than a 2-class model (Table 2). As such, the respective model was not explored further. Although the mean IQs for children with FASD and for typically developing control children were found to be significantly different (87.2 [SD = 10.2] vs. 106.4 [SD = 12.9], respectively; *p* < 0.001), the post-hoc analysis demonstrated that these two groups of children could not be differentiated based on IQ only.

#### Analysis 2: children with FASD vs. typically developing control children and children with other neurodevelopmental disorders

**Fig. 1:**
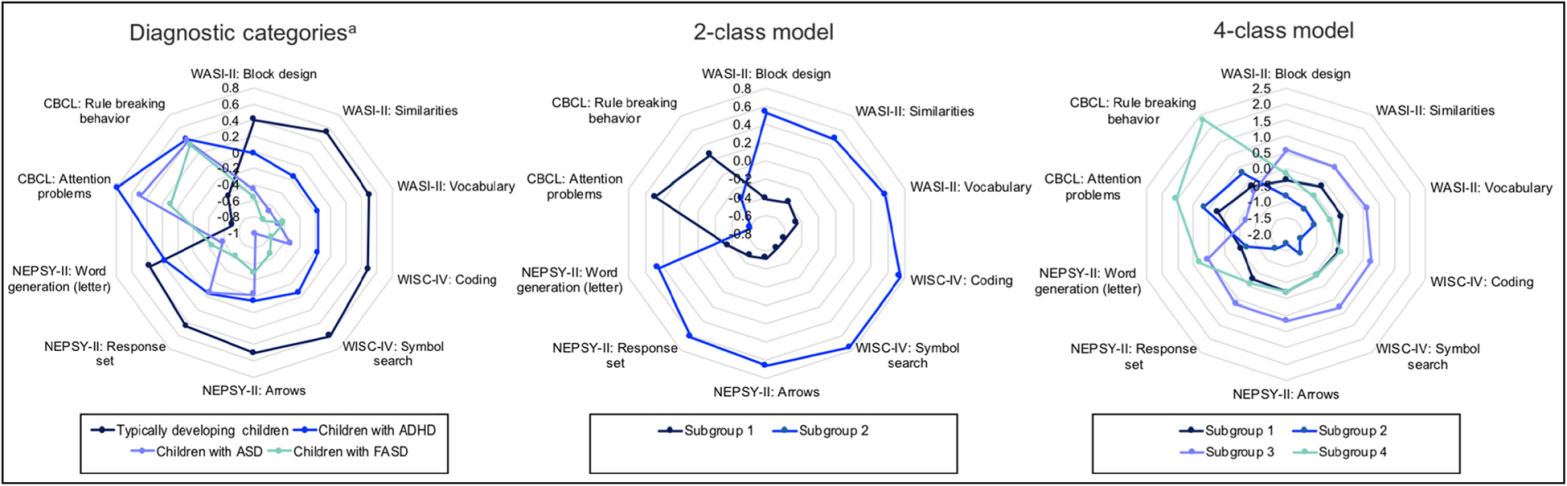
Mean scores for each diagnostic category and subgroup in the 2- and 4-class models in analysis 2. ADHD: Attention Deficit Hyperactivity Disorder; ASD: Autism Spectrum Disorder; CBCL: Child Behavior Checklist; FASD: Fetal Alcohol Spectrum Disorder; NEPSY-II: NEPSY, Second Edition; WASI-II: Wechsler Abbreviated Scales of Intelligence, Second Edition; WISC-IV: Wechsler Intelligence Scale for Children, Fourth Edition. ^a^Children with comorbid diagnoses are not included. *Note.* All scores are presented as z-scores. In the 2-class model, subgroup 1 is comprised of 20 children with FASD, 22 children with other neurodevelopmental disorders, and six typically developing children; and subgroup 2 is comprised of one child with FASD, six children with other neurodevelopmental disorders, and 31 typically developing children. In the 4-class model, subgroup 1 is comprised of 13 children with FASD, 15 children with other neurodevelopmental disorders, and six typically developing children; subgroup 2 is comprised of five children with FASD and four children with other neurodevelopmental disorders; subgroup 3 is comprised of five children with other neurodevelopmental disorders and 31 typically developing children; and subgroup 4 is comprised of three children with FASD and four children with other neurodevelopmental disorders

A 2-class model best described the overall sample (Table 2), and correctly identified 83.8% (31 out of 37 [specificity]; 95% CI: 71.0–94.3%) of typically developing children as not having either FASD or other neurodevelopmental disorders (Table 5). However, the 2-class model was only able to correctly identify 56.9% (37 out of 65 [specificity]; 95% CI: 45.1–69.2%) of children with other neurodevelopmental disorders and typically developing children as not having FASD. The 2-class model resulted in almost all children with FASD (20 out of 21) and considerably more children with other neurodevelopmental disorders (22 out of 28) being assigned to subgroup 1, and significantly more typically developing children (31 out of 37) being assigned to subgroup 2 (Table 5). As would be expected, participants in subgroup 1 performed worse than participants in subgroup 2 for each of the eight observed variables derived from the neurodevelopmental subtests and scored higher on the two observed variables derived from the CBCL (Table 3 and Fig. 1).

**Table 5:** Number of participants assigned to each subgroup and the classification function of the 2- and 4-class models in analysis 2, and the 2-class model in the sensitivity analysis

Group assignment; Classification function (%)	Main Analysis						Sensitivity Analysis		
	2-class model			4-class model			2-class model		
	Children with FASD (n)	Children with other neuro developmental disorders (n)	Typically developing control children (n)	Children with FASD (n)	Children with other neuro developmental disorders (n)	Typically developing control children (n)	Children with FASD (n)	Children with ADHD (n)	Typically developing control children (n)
Subgroup 1	20	22	6	13	15	6	20	16	6
Subgroup 2	1	6	31	5	4	0	1	6	31
Subgroup 3	–	–	–	0	5	31	–	–	–
Subgroup 4	–	–	–	3	4	0	–	–	–
Children with FASD vs. Typically developing control children and children with other neurodevelopmental disorders^a^									
Sensitivity (95% CI)	95.2 (82.6–100.0)^b^			100.0 (n/a)^c^			95.2 (84.6–100.0)^d^		
Specificity (95% CI)	56.9 (45.1–69.2)^b^			55.4 (43.5–67.2)^c^			62.7 (50.0–74.6)^d^		
PPV (95% CI)	41.7 (28.2–55.3)^b^			42.0 (29.0–56.0)^c^			47.6 (32.0–62.5)^d^		
NPV (95% CI)	97.4 (91.4–100.0)^b^			100.0 (n/a)^c^			97.4 (91.4–100.0)^d^		
Children with FASD and children with other neurodevelopmental disorders^a^ vs. Typically developing control children									
Sensitivity (95% CI)	85.7 (73.5–95.5)^e^			89.8 (81.1–97.7)^f^			83.7 (72.1–94.1)^g^		
Specificity (95% CI)	83.8 (71.0–94.3)^e^			83.8 (71.4–94.7)^f^			83.8 (71.0–94.7)^g^		
PPV (95% CI)	87.5 (78.2–95.7)^e^			86.1 (75.0–97.1)^f^			85.7 (74.3–95.3)^g^		
NPV (95% CI)	81.6 (68.4–94.6)^e^			88.0 (79.1–95.9)^f^			81.6 (68.6–93.1)^g^		

*ADHD* Attention deficit hyperactivity disorder, *CI* Confidence interval, *FASD* Fetal alcohol spectrum disorder, *n/a* Not applicable, *NPV* Negative predictive value, *PPV* Positive predictive value

^a^Sensitivity analysis excludes children with Autism Spectrum Disorder from the group of children with other neurodevelopmental disorders

^b^Assuming subgroup 1 is reflective of the neurodevelopmental profile of FASD and subgroup 2 is reflective of typically developing control children and children with other neurodevelopmental disorders

^c^Assuming subgroup 1, 2 and 4 are reflective of the neurodevelopmental profile of FASD and subgroup 3 is reflective of typically developing control children and children with other neurodevelopmental disorders

^d^Assuming subgroup 1 is reflective of the neurodevelopmental profile of FASD and subgroup 2 is reflective of typically developing control children and children with ADHD

^e^Assuming subgroup 1 is reflective of the neurodevelopmental profile of FASD and children with other neurodevelopmental disorders and subgroup 2 is reflective of typically developing control children

^f^Assuming subgroup 1,2 and 4 are reflective of the neurodevelopmental profile of FASD and children with other neurodevelopmental disorders and subgroup 2 is reflective of typically developing control children

^g^Assuming subgroup 1 is reflective of the neurodevelopmental profile of FASD and children with ADHD and subgroup 2 is reflective of typically developing control children

The 4-class model resulted in all 21 children with FASD and most children with other neurodevelopmental disorders (23 out of 28) being assigned to subgroup 1, 2, or 4, and the majority of typically developing children (31 out of 37) being assigned to subgroup 3. However, the 4-class model did not produce subgroups that were reflective of the diagnostic constructs represented in the sample (see Fig. 1); the model correctly identified only 55.4% (36 out of 65 [specificity]; 95% CI: 43.5–67.2%) of children with other neurodevelopmental disorders and typically developing children as not having FASD.

##### Sensitivity analysis

A 2-class model best fit the data (Table 2). However, the 2-class model was only able to correctly identify 62.7% (37 out of 59 [specificity]; 95% CI: 50.0–74.6%) of children with ADHD and typically developing children as not having FASD (Table 5). Thus, although children with FASD performed most similarly to children with ASD on the neurodevelopmental subtest included in the profile (Fig. 1), including a small number of children with ASD did not appear to negatively influence the ability of the neurodevelopmental profile to differentiate children with FASD from children with other neurodevelopmental disorders.

## Discussion

Although the neurodevelopmental profile identified was sensitive to FASD, it was not specific to FASD, suggesting that a neurodevelopmental profile that can differentiate children with FASD from children with other neurodevelopmental disorders may not exist. However, the findings are limited by the measures used in the analyses, as the inclusion of additional measures may have resulted in a more specific FASD neurodevelopmental profile. Also, data on the use of psychotropic medications were not available. Given that such medications are intended to alter brain function, their use could have impacted the results of the current study. Despite the relatively small sample size, albeit sufficient [31], the latent profile analyses did produce statistically and clinically significant results. Given the few cases of ASD, it was not possible to provide classification results for ASD specifically. Even though the 4-class model did result in children with neurodevelopmental disorders (including FASD) being broken down into subgroups, they were not grouped according to their diagnostic categories. It should be acknowledged that it is possible that the subgroupings could have been an artifact of the methodology used, as the primary goal of LPA is to maximize the homogeneity within subgroups and the heterogeneity between them.

The findings of the current study are in line with those of Mattson and colleagues [32], who were able to demonstrate that a set of neurodevelopmental tests measuring executive function, attention, and visual and spatial memory could differentiate between individuals with FASD and individuals not exposed to alcohol prenatally; however, when using a clinical comparative group, the profile was more accurate at identifying individuals with ADHD than individuals with FASD. Unlike previous studies seeking to identify a unique neurodevelopmental profile of FASD, the current study used a population-based sample of children with FASD – the sample was drawn from a cross-sectional, population-based study that utilized active case ascertainment (the gold standard [33]) to identify cases of FASD. This is also the first study to analyze and incorporate both behavioral observations/ratings of parents and performance-based measures of neurodevelopment when seeking to identify a neurodevelopmental profile of FASD.

Although prenatal alcohol exposure is a necessary cause of FASD, the genetic etiology of FASD remains unknown. Advances in the understanding of genetics and its role in neurodevelopmental disorder risk have created a paradigm shift, such that neurodevelopmental disorders are no longer viewed as having a psychogenic etiology but rather a genetic etiology (see for example, Glessner et al. [34]). Prenatal alcohol exposure leads to epigenetic changes (i.e., altered gene expression) [35]. These changes may contribute to the spectrum of effects and different phenotypes observed in children with FASD [35]. The discovery of reliable genetic and epigenetic markers for FASD would have significant implications for its diagnosis. Such investigations should not be restricted to FASD, but rather include all neurodevelopmental disorders, as this may lead to current categorical classifications of neurodevelopmental disorders being redefined to be more reflective of biologically homogeneous groups [36]. Accordingly, future studies should explore whether neurodevelopmental data combined with genetic and epigenetic data would produce a profile able to diagnose and differentiate FASD from other neurodevelopmental disorders.

## Data Availability

The datasets used and/or analysed during the current study are available from the corresponding author on reasonable request.

## Abbreviations

ADHD: Attention Deficit Hyperactivity Disorder
AIC: Akaike Information Criterion
ARND: Alcohol-Related Neurodevelopmental Disorder
ASD: Autism Spectrum Disorder
BIC: Bayesian Information Criterion
CBCL: Child Behavior Checklist
CI: Confidence interval
DSM-5: Diagnostic and Statistical Manual of Mental Disorders, Fifth Edition
FAS: Fetal Alcohol Syndrome
FASD: Fetal Alcohol Spectrum Disorder
LLV: Log-Likelihood Value
LPA: Latent profile analysis
ND-PAE: Neurobehavioral Disorder Associated with Prenatal Alcohol Exposure
NEPSY-II: NEPSY, Second Edition
NPV: Negative predictive value
OFC: Occipitofrontal circumference
pFAS: Partial Fetal Alcohol Syndrome
PFL: Palpebral fissure length
PPV: Positive predictive value
SD: Standard deviation
WASI-II: Wechsler Abbreviated Scales of Intelligence, Second Edition
WISC-IV: Wechsler Intelligence Scale for Children, Fourth Edition
